# Practice, enablers and barriers of health information system accountability framework in Northwest Ethiopia 2023

**DOI:** 10.1186/s12911-025-02942-8

**Published:** 2025-03-03

**Authors:** Biniyam Tilahun, Berhanu Fikadie Endehabtu, Amare Minyihun, Tajebew Zayede, Adane Nigusie, Asmamaw Atnafu, Lemma Derseh, Tesfahun Hialemarima, Getasew Amare

**Affiliations:** 1 Department of Health Informatics, Institute of Public Health, College of Medicine and Health Sciences, University of Gondar, P.O.BOX: 196, Gondar, Ethiopia; 2 Department of Health Systems and Policy, Institute of Public Health, College of Medicine and Health Sciences, University of Gondar, Gondar, Ethiopia; 3 Department of Health Education and Behavioral Science, Institute of Public Health, College of Medicine and Health Sciences, University of Gondar, Gondar, Ethiopia; 4 Department of Epidemiology and Biostatistics, Institute of Public Health, College of Medicine and Health Sciences, University of Gondar, Gondar, Ethiopia; 5 Center for Digital Health and Implementation Science (CDHI), University of Gondar, Gondar, Ethiopia; 6 Amhara Public Health Institute, Bahir Dar, Ethiopia

**Keywords:** Health information system, Accountability framework, Northwest Ethiopia

## Abstract

**Background:**

The government of Ethiopia has designed different initiatives for the Health Information Systems (HIS), including an Information Revolution (IR) transformation agenda by 2015. Various interventions and working documents have also been developed and implemented targeting the different aspects of the HIS program. However, there is no nationally designed accountability framework to govern HIS activities. Besides, how health institutions follow and monitor HIS activities is unknown. Therefore, this study aimed to assess the practice and barriers of HIS accountability framework at the selected public health institutions.

**Method:**

A descriptive qualitative study design was employed from June 05 to July 12, 2023. Purposively selected informants from public health institutions were recruited for key informant interviews. A prepared pilot-tested semi-structured interview guide was used. The conventional content approach was used to summarize and synthesize the information explored.

**Findings:**

The study revealed that most respondents described the concept and advantages of the HIS accountability framework in different ways. The participants believed the HIS accountability framework would help to govern and manage behavioral-related HIS challenges. It was indicated that the framework will help to control the recurrence of HIS errors, enhance the commitment and adherence of health professionals, and improve data handover practice, data security and privacy, data quality, informed decision, and finality quality of care. Lack of national guidelines on the HIS accountability framework, the poor culture of accountability, multiple responsibilities and workload, high staff and leadership turnover, lack of motivation, and security problems were stated barriers to implementation of the HIS accountability framework. It was suggested to create a conducive work environment, engage health professionals and other actors during the intervention development, build the skills on HIS leadership, and have the national HIS accountability framework document to implement the intervention effectively.

**Conclusions:**

Even if there is a better understanding of the concept and advantages of the HIS accountability framework, its practice across the system is limited. It would be better to design the HIS accountability framework using a human-centered design/approach by engaging the key HIS actors and understanding their working environment.

**Supplementary Information:**

The online version contains supplementary material available at 10.1186/s12911-025-02942-8.

## Background

The government of Ethiopia has been investing a lot in expanding and strengthening the health system across the regions to improve access to primary health care [1]. Though progressive efforts were made to improve the healthcare system and health service delivery, the quality data production and use of data for planning and informed decision-making remains a key challenge [2]. Safe and reliable healthcare is also challenged due to limitations related to the quality of health data production and use [3].

Over the past several years, attempts have been made to overcome the challenges related to healthcare data quality and use [2]. The HIS gets national priority and is one of the key transformation agendas in the Ethiopian Health Sector Transformation Plan (HSTP) [4, 5]. Infrastructure development and preparation of different implementation manuals and procedures were done extensively to enhance the quality of data production and informed decision-making [5, 6]. In addition, in-service and pre-service capacity building and health professionals’ training on the HIS program were done [6, 7]. The development health informatics technicians (HIT) with the role of data entry, analysis, reporting, and overseeing data management task at the health institutions was another intervention done by the health system and supporting partners [8].

The concept of accountability framework involves taking ownership of assigned duties and being accountable for fulfilling them by reporting on their progress. It plays a vital role in defining roles, responsibilities, and potential consequences for failing to adhere to established protocols [9]. Implementation of accountability systems allows employees to be productive and efficient in their work, resulting in improved quality of health services and good performance [10]. Though the HIS accountability framework is recommended to improve data quality and use, its usability is limited [11]. Moreover, its experiences and enactment are limited, though there is a global call to improve the practice of accountability in the health system [12]. The experiences and daily practices of managers, healthcare providers, and community agents towards accountability framework are poorly understood in Ethiopia’s primary healthcare system [12]. Besides, how the HIS activities are monitored, governed, and controlled across different health system levels is not well studied. Therefore, the study aimed to explore the practice, enablers and barriers of HIS accountability framework implementation and use at the public health institutions of northwest Ethiopia.

## Method and approaches

### Research team and reflexivity

Key Informant Interviews were conducted by a team of experts from the Institute of Public Health at the University of Gondar, including GA, AN, BFE, TZ, and AM. These individuals possess extensive knowledge and experience in gathering and analyzing qualitative research data. The investigators AN, BT, AA, TH, and LD held PhDs, while GA, BFE, TZ, and AM had Master’s degrees in Public Health (MPH). All investigators were male and worked as researchers and lecturers during the study. Prior to the commencement of the study, the investigators made efforts to build rapport with the participants. They disclosed their identities, explained the study’s objectives, and provided a rationale for conducting the research to ensure transparency and open communication.

### Study design

A descriptive qualitative study design was employed from January 2023 to July 2023. We chose this specific study design because of our unwavering dedication to producing high-quality research and the accessibility of our methodology for other researchers. Despite our extensive experience in qualitative research, we acknowledge the unique benefits of this method in thoroughly exploring the factors that support or hinder the accountability framework in HIS. By adhering strictly to qualitative research principles and ensuring that our findings are easily understandable by different stakeholders, our aim is to uncover insights that can lead to significant improvements in the governance of HIS activities.

The descriptive qualitative study design was chosen because of its ability to provide a comprehensive understanding of the complex issues being studied, its adaptable nature in capturing changing processes, and its accessibility to various stakeholders [13, 14]. By harnessing the strengths of this methodology, we hope to gain valuable insights that can drive progress in addressing the challenges and opportunities within the HIS accountability framework.

### Theoretical framework

#### Methodological orientation and theory

The descriptive qualitative study is highly suitable for researching health environments as it yields straightforward answers to queries regarding people’s sentiments towards a specific space, the motivations behind utilizing certain aspects of the space, the demographics of individuals using specific services or functions within the space, as well as the factors that either facilitate or impede usage [15]. Qualitative descriptive designs are characterized by their methodological diversity and are rooted in the inherent principles of constructivist investigation. This approach is particularly advantageous for healthcare environment designers, practitioners, and health sciences researchers as it elicits detailed insights from the perspective of the subjects involved [16].

### Participant selection and sample description

This study included diverse participants from various levels of the health system, such as service providers, HMIS officers, health facility managers, Woreda health office leaders, planning officers, M&E officers, and zonal health department heads. Intensity purposive sampling was used to select 27 key informants (KIIs) based on their expertise, roles, and active involvement in HIS activities. The selection ensured diversity in perspectives, avoiding bias while capturing a comprehensive understanding of HIS accountability frameworks. Participants were approached through face-to-face interviews using open-ended questionnaires, with recruitment continuing until data saturation was reached.

### Saturation in qualitative data collection

In qualitative studies, saturation is a key factor to consider. It’s the point where no new ideas or understandings come out of gathering more data [17, 18]. To determine saturation, we continuously checked emerging themes and insights from interviews and surveys during data collection and analysis. We kept collecting data until we reached thematic saturation, meaning no fresh information or perspectives were emerging. This iterative method guaranteed that the qualitative data we gathered for our study was sufficient and comprehensive.

### The setting of the study area

**Fig. 1:**
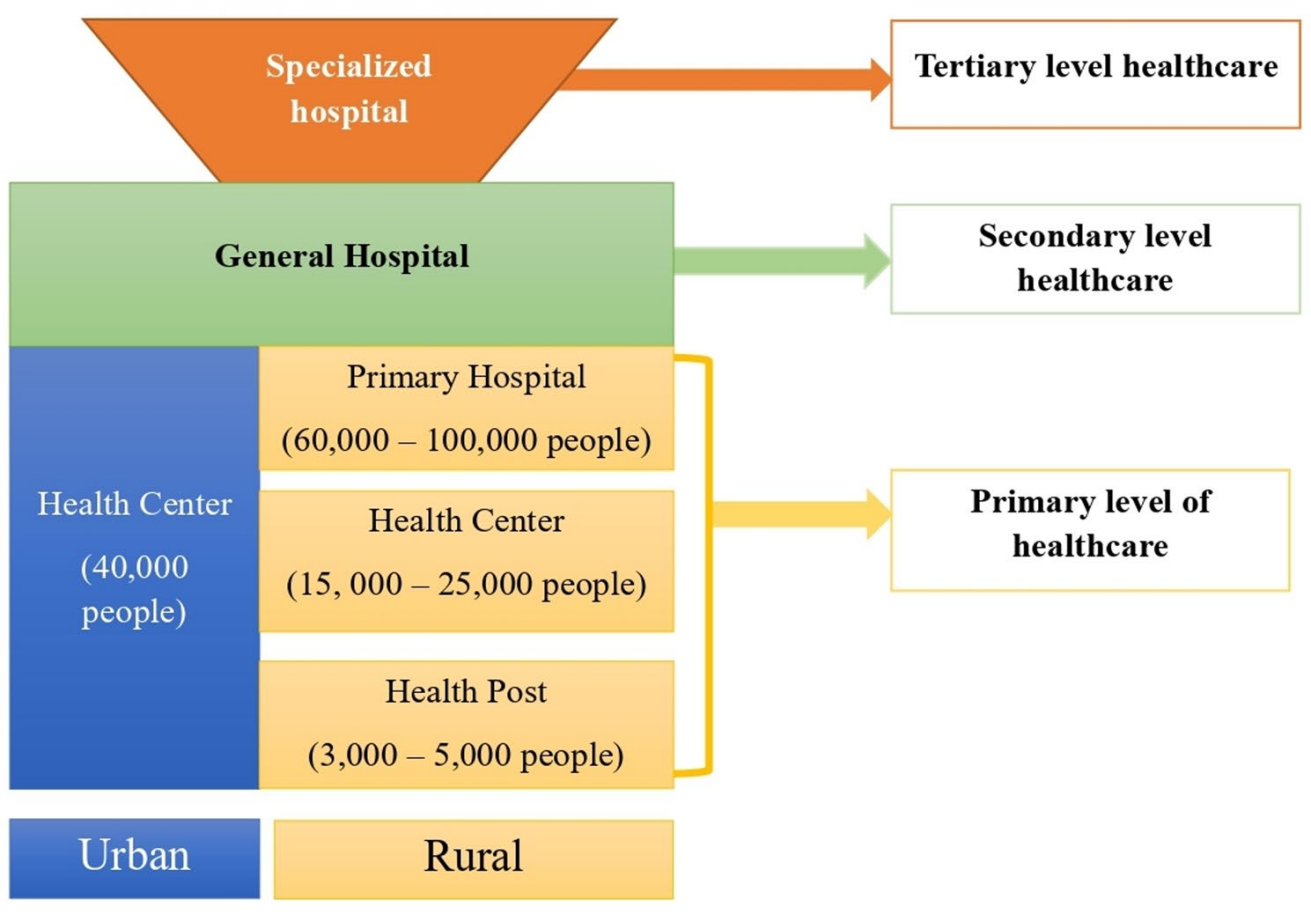
Health system tiers of Ethiopia (1). Ethiopian M. Ethiopian health sector transformation plan I. 2015

The healthcare system in Ethiopia is organized into a three-tier structure, encompassing primary, secondary, and tertiary levels of healthcare services. At the primary level, there is the primary health care unit, which includes health centers, five health posts within each health center, and a primary hospital. Each health post is responsible for a population of 3,000–5,000 individuals, ensuring access to basic preventive and promotive healthcare services. Both preventive and curative services are provided at the health centers, while the primary hospital goes a step further by offering emergency surgical procedures like caesarean section and blood transfusions. Moving on to the secondary level of care, there are general hospitals that also serve as referral centers for primary hospitals. These facilities provide a wider range of medical services to cater to more complex health issues. Finally, at the tertiary level of care, there are federally-run specialized hospitals and university hospitals that offer advanced medical treatments and specialized care for complex and rare medical conditions. These hospitals play a crucial role in serving as centers of excellence in healthcare delivery and medical research. Figure 1. The study was conducted at randomly selected public health institutions in the central Gondar zone and Gondar city administration which included health centers, hospitals, woreda health offices and zonal health departments.

### Presence of non-participants

There are no other participants in the study except the participants and researchers.

### Data collection

We used a piloted and semi-structured interview guide prepared in English and translated into an Amharic tool to elicit data details through probes. The interview guide for the KIIs was designed based on literature related to the main research questions. The interview guides included eight broad questions with suggested probes for the KIIs (Supplementary file 01). The guide was developed to capture participants’ views on the understanding, practice, enablers, and barriers to the HIS accountability framework.

The interview questionnaire was crafted by considering the research objectives and pertinent literature on the implementation, barriers, and facilitators of HIS accountability framework and governance. It consisted of open-ended queries carefully created to draw out comprehensive answers from respondents regarding their encounters and viewpoints on the practice, barriers and enablers concerning HIS accountability framework. Additional probing questions were utilized to delve deeper into emerging themes. Besides, the study was conducted in a secure setting through a face-to-face interviews.

The number of participants interviewed was determined by information saturation, which means that the information generated from repeated interviews becomes saturated. Thus, a purposively selected 27 KII interviews were conducted. Once recruited and agreed to participate, consent was taken for the interview. The research used audio recording to collect the data.

During and post the interview, the investigators took detailed field notes to capture essential contextual details. These notes provided a deeper understanding of the statements made by the participants, enhancing the recorded and transcribed information. Additionally, the field notes helped in identifying speakers when their voices sounded alike, as well as recording non-verbal cues such as body language, facial expressions, pauses, and eye contact. These detailed observations enriched the overall interpretation of the conversation. The interviews varied in length, taking from 28 to 55 min. On average, the interviews lasted about 40.4 min, with a standard error of 1.5.

### Data analysis

Two well-experienced researchers performed the data analysis. The researchers were trained on qualitative data analysis methods and using Open Code 4.03 software for data management [19]. Conventional content analysis was applied to analyze the data. The conventional content analysis describes a phenomenon where existing research and theory are limited [20]. Notes are made, and codes are categorized. Steps for the data analysis: (1) a coding manual containing a beginning list of codes derived from the theoretical framework and literature and preliminary data analysis was developed before initiating data collection. Codes are action-oriented words or labels assigned to designated portions (chunks or meaning units) of text reflecting themes or topics that occur with regularity. (2) Each transcribed document was formatted with wide right margins, allowing the investigator to apply codes and generate marginal remarks by hand. (3) The investigator took sentences or paragraphs in the transcripts and divided them into meaning units, segments of text containing a single idea. (4) Conceptually similar codes were organized into categories (coding groups of coded themes that were increasingly abstract) by revisiting the theory framing the study. (5) During this analysis phase, pattern codes were revised and redefined in the coding manual, and exemplars were used to clarify the understanding of each code. (6) Analytic memos: “brief or extended narrative that documents the researcher’s reflections and thinking processes about the data.” Memos aided in data reduction by tying together different pieces of data into conceptual clusters. Memos were personal, methodological, or substantive. (7) Data displays (matrices) or visual representations containing concepts or variables helped analyze the data. (8) Finally, the data are represented in a creative but rigorous way that is judged to fit the findings best.

Two individuals conducted the coding process separately after thoroughly reviewing the transcribed document multiple times. Every interview conducted and field notes taken were faithfully transcribed into Amharic, the local language, and then translated into English. These translated transcripts were then inputted into Open Code 4.03 software for coding purposes. The study employed a structured approach involving four key phases to analyze the data: (1) getting familiar with the data, (2) Reviewing the research objectives, (3) developing a framework, and (4) Identifying patterns and connections. The core themes were derived from the inherent meaning of the categories. The researchers rigorously verified the emerging themes by comparing them with relevant quotes in each theme. The results were presented through a detailed explanation and interpretation of the themes’ significance. Participants’ direct quotes were incorporated in the findings to offer vivid insights to the readers. The analysis followed an inductive method, where the research team engaged in a thorough discussion to uncover themes from the data. The findings of the study were further detailed according to the established guidelines outlined in the consolidated criteria for reporting qualitative research (COREQ) guidelines [21] (Supplementary file 02). The theme and sub-themes were derived from the data. Open code 4.03 software was used to manage the data [22].

### Trustworthiness

The credibility of any research study was upheld through the credibility of its findings. Professionals meticulously examined the protocols to guarantee the integrity of the data, while a trial run was conducted to assess the coherence of the questions and their alignment with cultural norms. The researchers employed straightforward language and explanations during interviews. An audit trail method was recognized as essential to validate the precision and trustworthiness of the outcomes. Principal investigators engaged extensively with participants and diligently observed them throughout the research process. Additionally, an external audit was conducted to validate the accuracy of the conclusions and to affirm that they were substantiated by the gathered data.

### Findings

#### Participant characteristics

In this study, 27 respondents participated through KIIs. Among the participants, six were female, and the rest were male. Besides, 22 respondents were from the health centers, and six were from the woreda health offices. Most participants have an overall experience of more than ten years and have worked at least four years at their current position. Most participants were HMIS officers and facility heads and had a BSc level of education. (Table 1).

**Table 1:** Socio-demographic characteristics of the study participants

No	Characteristics	Level	Frequency	Relative frequency
1.	Age	<= 30 years	7	25.9
		30–35 years	11	40.7
		> 35 years	9	33.3
2.	Sex	Male	16	59.3
		Female	11	40.7
3.	Institution	Health centers	14	51.9
		Hospitals	3	11.1
		Woreda health offices	6	22.2
		Zonal health department	2	7.4
		City administration	2	7.4
4.	Total years of experience	<= 5 years	4	14.8
		5–10 years	12	44.4
		> 10 years	11	40.7
5.	Total years of experience in the current position	<= 5 years	17	63.0
		> 5 years	10	37.0
6.	Position	HMIS officers	12	44.4
		Service providers	4	14.8
		M&E officer	2	7.4
		Plan officer	2	7.4
		Facility heads	7	25.9
7.	Level of education	Masters of Public Health (MPH)	6	22.2
		Bachelor of Science (BSc)	13	48.1
		Diploma	8	29.6
Total			27	100

### Main findings

The study identified the understanding, perceived benefits, enablers, and barriers of HIS accountability framework implementation and respondents’ suggestions to design and implement an effective HIS accountability framework. The findings are presented into six themes: understanding of HIS accountability framework, perceived benefit, implementation, enablers, barriers, and suggestions. Table 2 summarizes the synthesized themes and key points of the study.

**Table 2:** Summary of themes, sub-themes, codes and sample quotes

Themes	Sub-themes	Codes	Sample quote
Understanding towards HIS accountability framework	Guidance and regulation	• System of controlling HIS activities using governing protocols	“ *HIS accountability framework is a system to oversee and regulate the HIS activities based on the assigned HIS roles and responsibilities”*
		• A system to ensure accountability for HIS roles and responsibilities	
		• A system to ensure being answerable for the HIS related work results	
		• A system to take administrative and corrective actions for the HIS activity results	
		• Regulatory means to monitor and control HIS activities	
		• Procedural standard to govern/cascade HIS activities	
Perceived advantage of HIS accountability framework	Improving behavior towards data management	• To manage the behavioral problems towards HIS practice	*“If the HIS accountability framework is implemented*,* it will govern the behavioral-related problems of the health professionals and be used to manage and control the recurrence of HIS-related errors based on the tangible guideline or protocol.”*
		• To enhance the adherence of health professionals for the HIS activities	
		• To enhance the commitment and sense of ownership of health professionals	
	Improving data quality and data use practice	• To enhance data handover and sharing practice	*“HIS accountability framework is an ideal system to ensure the data quality which is appropriate recording and reporting practice and to use it for different program and clinical decisions”*
		• To enhance data security and privacy	
		• To ensure to data timeliness, completeness, and consistency	
		• To improve informed decision making practice & quality of care	
		• To have valid research output	
	Improving HIS leadership practice	• To effectively apply HIS leadership practices	*“If the HIS accountability framework is introduced at the institutions*,* it will bring a good culture to give specific and targeted feedback to the implementers*,* enhancing the health professionals’ sense of ownership of the HIS data”*
Implementation of HIS accountability framework	Current practice of HIS accountability framework	• No written system to govern the HIS activities	*“…In my experience*,* I have not seen any experience of applying accountability system to oversee the activities of HIS”*
		• No Accountability framework practice	
	HIS implementation M&E platforms	• HIS specific internal supervisions	*“We have a PMT comprised of various unit leads*,* responsible for overseeing the practice of health data and program performance activities. This team serves as a valuable M&E platform to assess the quality of data and program effectiveness on a monthly basis.”*
		• Performance Review Meeting (PMT)	
		• HMIS task audit	
		• Monthly HIS monitoring	
		• Supportive supervision	
		• Regular feedback	
		• Review meetings	
Enablers of HIS accountability framework	Policy and practice	• National priority	*“HIS is the current priority area and transformation agenda of the health system which is one potential opportunity to better implement the HIS accountability framework initiative”*
		• Partner engagement	
		• Progress in HIS program	
	Tools and infrastructure	• Digitization	*“In the HIS program*,* digital systems are deployed and used for different purposes like DHIS2*,* eCHIS*,* mBrana*,* and Dagu. Applying these systems motivates health professionals to adhere to and value health data properly. This will be a good factor for effectively implementing the HIS accountability framework”*
		• Standardization of tools	
	Skill and capacity	• Experience of implementing the HIS activities	*“HIS capacity building is highly provided in the in-service and pre-service modalities which gives the opportunity to have better number of health information experts. This would be a good facilitator the better implement the accountability framework.*
		• Skill and number of HIS professionals	
Barriers of HIS accountability framework	Poor culture of accountability	• Poor culture of accountability practice/system	*“The culture and practice of making health professionals or other actors accountable for HIS related activities is very limited which will be a challenge to easily implement the HIS accountability framework”*
	Poor attitude towards health data	• Poor attitude for HIS from the leadership	*“Weak leadership commitment throughout the system poses a significant challenge in effectively implementing the HIS accountability framework. There is a tendency for leaders to prioritize politically motivated tasks and activities over routine data related tasks"*
		• Poor leadership commitment	
	High turn over	• Leadership turnover	*“High turnover of staff can hinder the effective execution of the HIS accountability framework*,* as experienced personnel depart and are replaced by new individuals who may lack familiarity with tasks associated with the HIS”*
		• Staff turnover	
	Poor infrastructure and guideline	• Lack of national guideline	*“There is no national governance to be used as a benchmark for the health institutions*,* and the culture and experience of practicing accountability is limited. This could alter the effective implementation of the HIS accountability framework.”*
		• Poor infrastructure for HIS supplies	
	High workload	• Multiple responsibility and task load	*“…staffs are working in a busy schedule: unless we manage the human resource need at each facility; it is difficult to design an accountability framework and order them to be governed by that framework.”*
		• Multiple recording tools	
	Insufficient knowledge and skill	• Skill and knowledge gap on HIS	*“…skill and capacity building is not full cascaded and provided which would another potential challenge in implementing the HIS accountability framework”*
		• Poor leadership skill	
	Security treat	• Political instability/insecurity	*“In the current condition*,* the health professionals are unstable and worry about other security issues. Besides*,* due to the interruption of the internet network*,* there is no means to follow and monitor the HIS tasks face to face and virtually. Due to this*,* it might be challenging to implement the framework in this condition"*
Suggestion to design effective HIS accountability framework	Participatory and engagement	• Engaging the staffs and case teams and other actors	“*As to me*,* most interventions have failed because they are not basing the real problem on the lower-level implementers. Therefore*,* engaging every actor and understanding their feelings and thoughts is very important for a successful intervention. If the engagement of the implementers develops the intervention*,* it will enhance the sense of ownership*,* and its adoption will be good.”*
		• Good to listen the saying, feeling, and emotion of the health professionals	
	Capacity building	• Build skill and knowledge on HIS and leadership	
	Priority	• Make HIS the political agenda	
	HIS supplies	• Fulfilling basic supplies and infrastructure of HIS activities	

### Understanding of HIS accountability framework

Awareness and understanding are very important for successfully implementing an intervention [23]. The majority of the participants explained HIS accountability framework as it is guidance and controlling system to govern the HIS task. Some respondents described HIS accountability as a system of controlling the HIS activities using administrative protocol.

*“… it is a management system to control the implementation of HIS related tasks using well defined governing protocols” (HMIS officer from the health facility)*.

Simultaneously, others described it as a method of overseeing and regulating HIS actions by defining the duties and obligations of healthcare providers.

“*HIS accountability framework is a system to oversee and regulate the HIS activities based on the assigned HIS roles and responsibilities” (Head of the health facility)*.

Respondents from the Zonal health department and Woreda health office better explain the HIS accountability framework. They describe the HIS accountability framework as a working document or system of ensuring the proper implementation of HIS-specific tasks. A respondent stated:

*“HIS accountability framework is a system of controlling HIS activities using a governing protocol that explains in detail the roles and responsibilities of health professionals and the consequences for not adhering to the protocol.” (M&E officer from the Zonal health department)*.

Other respondents explained that the HIS accountability framework is a regulatory and administrative document that ensures the proper implementation of HIS-specific activities by the implementers. A respondent stated:

*“HIS accountability framework is a governance document that clearly states what to do and not to do and makes answerable the implementers for their work results related to HIS activities” (HMIS officer from the woreda health office)*.

Another respondent also defines the HIS accountability framework as:

*“HIS accountability framework is a procedural standard that is used to govern or cascade the HIS activities…” (Head of the health center)*.

### Perceived advantage of HIS accountability framework

Perceived advantage is the benefit of an intervention stated from the target audience’s perspective. In this study, the perceived advantage has explored the benefit of the HIS accountability framework from the implementer’s perspective. The key informants explained the role of the HIS accountability framework for the HIS program and the health system in different ways. Under perceived advantage topic, improving behavior towards data management, improving data quality and data use practice, improving HIS leadership practice, improving HIS performance sustainability sub-topics were merged.

### Improving behavior towards data management

Many respondents revealed that developing and implementing the HIS accountability framework will improve the practice of HIS activities as per the national standard by managing the behavioral problems of implementers. By applying administrative actions, they stated that the intervention would help control recurrent HIS errors due to negligence and poor attitude. A respondent stated

*“If the HIS accountability framework is implemented*,* it will govern the behavioral-related problems of the health professionals and be used to manage and control the recurrence of HIS-related errors based on the tangible guideline or protocol.” (HMIS officer from the health center)*.

Other groups of respondents stated the HIS accountability framework’s role in improving health professionals’ adherence and commitment to HIS activities. They narrated that the HIS accountability framework will be used as a good alarming/stimuli intervention to take corrective actions and to give recognition based on the HIS work results. A respondent stated:

*“HIS accountability framework will be used as a stick and carrot strategy to give recognition for good performances and take corrective action for the reverse one. This strategy will alarm health professionals and improve their commitment to HIS activities.” (Head of the health center)*.

### Improving data quality and data use practice

Besides, it was also explored that the HIS accountability framework intervention will help ensure health data quality, informed decision-making, local data use for patient clinical care and admin-level decisions, sustainability of HIS activities, and healthcare quality. It was stated as:

*“The HIS accountability framework will help to produce quality health data and enhance the local data usage for decision making and improving the service quality.” (Head of woreda health office)*.

It was also explored that the HIS accountability framework will bring a good culture and practice of health data handover and data sharing. The respondents also added the HIS governing protocol would give the facilities a legal framework to ensure data security and privacy. They stated that the facilities didn’t have any legal ground or framework to practice data handover during staff rations, transfers, and leaves due to the lack of a governing protocol. The informant stated

*“As you know*,* when there are staff transfers and rotations*,* there is the handover of different institutional resources*,* but even if health data is one of the key institutional resources*,* we didn’t have any system and experience for health data. So*,* this kind of governance framework will help bring this culture to the institutions.” (M&E officer from zonal health department)*.

The respondents also stated the advantage of the HIS accountability framework in terms of its potential in improving the quality data generation and using it for local decision making. It was explained that HIS accountability framework would ensure the timely and valid data production for right time decision making. The respondent stated:

*“HIS accountability framework is an ideal system to ensure the data quality which is appropriate recording and reporting practice and to use it for different program and clinical decisions” (Service provider from the health facility)*.

It was also stated that the HIS accountability framework will help with quality data generation, storage, and legal data sharing for intended users. This helps to provide quality data for different researchers and helps to generate valid research output. A respondent stated:

*“As you know*,* most health researchers use patient level and aggregate secondary data as a data source for their objectives. But*,* most of the time*,* the secondary data sets are either incomplete or inaccurate*,* affecting the reliability and validity of the research findings. So*,* the HIS accountability framework will help to address this problem and to generate valid research findings” (M&E officer from zonal health department)*.

### Improving HIS leadership practice

Other respondents also described the role of the HIS accountability framework as it will help to objectively monitor and control HIS tasks and give practical feedback to the practitioners. They stated that the accountability framework is one crucial organizational input for implementing leadership practices effectively. A key informant said:

*“If the HIS accountability framework is introduced at the institutions*,* it will bring a good culture to give specific and targeted feedback to the implementers*,* enhancing the health professionals’ sense of ownership of the HIS data.” (HMIS officer from the Hospital)*.

Another respondent added

*“Without an accountability framework*,* it is difficult to say we are applying the proper leadership practices. Even if it is not the only input*,* HIS accountability framework is one key factor for effective HIS leadership” (M&E officer from the zonal health department)*.

### Implementation of HIS accountability framework

Practice is the application of an intervention for the sake of achieving a desired target or objective. The practice of the HIS accountability framework was assessed through respondents’ experience at their respective institutions on applying the intervention to implement HIS tasks. Under the practice of HIS accountability framework HIS accountability practice and HIS activities M&E platforms were identified as sub-themes.

#### Current practice of HIS accountability framework

The majority of the respondents stated they didn’t have a HIS-specific accountability framework document prepared by their institution or forwarded from the higher officials. A respondent stated:

*“…In my experience*,* I have not seen any experience of applying accountability system to oversee the activities of HIS” (HMIS officer from the woreda health office)*.

Respondents also stated that as they didn’t have any written and guiding document aligned with accountability to monitor and evaluate the practice of HIS related tasks. It was explained as:

“*We didn’t have any written guiding framework developed to objectively monitor the HIS activities and ensuring accountability based on the work results” (M&E officer from zonal health department)*.

### HIS implementation M&E platforms

The assessment also explores different M&E platforms like HIS specific internal supervisions, Performance Review Meeting (PMT), HMIS task audit, Monthly HIS monitoring, Supportive supervision, Regular feedback, and Review meetings to monitor and follow HIS activities.

The respondents stated they use supportive supervision and internal HIS task audits as potential strategies to follow the HIS tasks and to provide corrective feedbacks. A key informant said:

*“We have a system of doing internal supervisions simply to check how HIS related activities are practiced and to give feedback using both oral and written forms” (Plan officer form the woreda health office)*.

Other respondents described using the Performance Monitoring Team (PMT) platform to monitor the key HIS activities and internal HMIS task audits by selected health professionals, followed by targeted and constructive feedback. These approaches are used to identify the gaps and to give corrective feedback, but they don’t have any role in taking any administrative actions. A respondent stated:

*“We have a PMT comprised of various unit leads*,* responsible for overseeing the practice of health data and program performance activities. This team serves as a valuable M&E platform to assess the quality of data and program effectiveness on a monthly basis” (HMIS officer from health center)*.

It was also stated that review meetings were used to assess the overall implementation, challenges and the way forward with the presence of different stakeholders. The platform described as means to discuss the challenges with the health system leadership and to set the common improving action plans. It was describes as:

*“We have a review meetings platform to review the HIS related activities from their timelines*,* completeness*,* consistency and their utilization for local decision making activities by engaging different actors” (M&E officer from zonal health department)*.

#### Enablers to implement HIS accountability framework

Enablers are the internal and external opportunities that enhance the successful implementation of the program or the intervention. Enablers in this study explored the potential opportunities that precipitate the implementation of an HIS accountability framework from the respondents’ perspective. The policy and practice, tools and infrastructure, and skill and capacity were among the sub-themes identified under the enablers section.

#### Policy and practice

Many key informants stated that national priority given to the HIS program, positive progress in HIS program performance, and engagement of different partners in the HIS program as the potential enablers for HIS program performance. They stated that IR is one of the top health sector transformation agendas which give high emphasis for health data management. A respondent stated:

*“HIS is the current priority area and transformation agenda of the health system which is one potential opportunity to better implement the HIS accountability framework initiative” (M&E officer from zonal health department)*.

Another respondent added:

*" The HIS program performance is improved in different parameters from time to time*,* and this is a good motivation factor to successfully adopt and implement HIS accountability framework” (Head of the health center)*.

#### Tools and infrastructure

The respondents also stated that the expansion and transformation of HIS into a digital system, partner engagement, and standardization of HIS tools are potential enablers. They noted the HIS program applies digital systems like District Health Information System (DHIS2), Electronic Community Health Information System (eCHIS), Dagu, mBrana, and others for data entry and management purposes. This enhances the attitude and value given to health data and the successful implementation of the intervention. A respondent stated:

*“In the HIS program*,* digital systems are deployed and used for different purposes like DHIS2*,* eCHIS*,* mBrana*,* and Dagu. Applying these systems motivates health professionals to adhere to and value health data properly. This will be a good factor for effectively implementing the HIS accountability framework.” (HMIS officer from Zonal health department)*.

Another respondent added:

*“There is consistency in the standardization of HIS tools*,* and partners are available to support us on different HIS activities like data quality*,* use*,* and eCHIS. This helps initiate and implement our institutions HIS accountability framework.” (HMIS officer from the Woreda health office)*.

### Skill and capacity

The assessment also explored that availability of potential skills and the capacity to implement HIS tasks is another opportunity to implement the HIS accountability framework. It was pointed out that the experience on HIS activities and the presence of sufficient HIS staff serve as an advantageous opportunity for the implementation of HIS accountability framework. The respondent stated:

“*HIS capacity building is highly provided in the in-service and pre-service modalities which gives the opportunity to have better number of health information experts. This would be a good facilitator the better implement the accountability framework” (Plan officer from the woreda health office)*.

### Barriers to implementing HIS accountability framework

Barriers are the challenges that hinder or deter an intervention’s successful implementation as expected. The barriers in this study explored the potential challenges that affect the implementation of the HIS accountability framework. Several barriers were discovered, primarily the poor culture of accountability, employee attitudes, frequent turnover in leadership and staff, limited infrastructure and guidelines, excessive workload, insufficient skill and capacity, and security measures.

### Poor culture of accountability

Respondents described the system of practicing and ensuring accountability as weak. It was explained that the culture of being accountable for assigned responsibility is not common practice and this will affect the effective implementation of the new HIS accountability framework. It was stated:

“*The culture and practice of making health professionals or other actors accountable for HIS related activities is very limited which will be a challenge to easily implement the HIS accountability framework” (HMIS focal from the health facility)*.

### Poor attitude towards health data

The study respondents also noted that the skill and commitment of leadership to initiate and implement this new initiative is a challenge. They added that there are leadership skill gaps in initiating and managing change, and the commitment of top leaders is also low. Leaders primarily emphasize those political activities that are reviewed by the cabinet. But, the HIS didn’t get adequate political attention. The respondent noted:

*“HIS activities are not getting political attention; due to this*,* the top leadership didn’t give attention to closely following the HIS activities and taking corrective actions. This problem might also affect the successful implementation of the initiative.” (HMIS officer from zonal health department)*.

The poor attitude towards HIS related activities by the health professionals was also stated other potential barrier to better implement HIS accountably framework. It was explained that:

*“… another challenge in effectively implementing HIS accountability framework would be the poor attitude towards health data from different perspectives especially at front line data producers” (HMIS focal from the health facility)*.

### High staff and leadership turnover

It has been mentioned that the other challenge in implementing the HIS accountability framework is the high turnover rate of staff and leadership. This turnover disrupts the continuity of experienced individuals within the organization, resulting in the need for constant updates and capacity building to fill the gaps. The respondent stated:

*“High turnover of staff can hinder the effective execution of the HIS accountability framework*,* as experienced personnel depart and are replaced by new individuals who may lack familiarity with tasks associated with the HIS” (M&E officer form the zonal health department)*.

#### Poor infrastructure and guideline

The assessment also explored lack national guidelines on HIS governance, burden of data recording with multiple recording tools and interruption in the availability of HIS tools as a potential barrier for HIS accountability framework implementation. The respondents stated lack of national accountability framework affects the initiation and implementation of the accountability system by due to absence of benchmarking document: The respondent stated:

*“There is no national governance to be used as a benchmark for the health institutions*,* and the culture and experience of practicing accountability is limited. This could alter the effective implementation of the HIS accountability framework.” (Head of the health center)*.

It was also stated that essential HIS supplies were not regularly available which also affects the proper implementation of HIS accountability framework. It was narrated:

*“… there is an interruption of HIS supplies availability like recording and tally tools which would affect the proper implementation of HIS accountability framework.” (Service provide from the health facility)*.

#### High workload and multiple responsibilities

The assessment also explored high workload and multiple responsibilities assigned on the health professionals. In addition, multiple responsibilities are assigned to single individuals, like coordinating different programs, making them overloaded and not focused on health data issues. The respondent stated:

*“…staffs are working in a busy schedule: unless we manage the human resource need at each facility; it is difficult to design an accountability framework and order them to be governed by that framework.” (Head of health center)*.

### Insufficient HIS knowledge and skill

The assessment revealed that sufficient capacity-building activities are not being effectively disseminated throughout all levels to adequately equip healthcare professionals with the necessary knowledge and skills to enhance their understanding of heath data practices. They added gaps on knowledge and skills would affect the implementation of HIS accountability framework. It was stated:

*“…skill and capacity building is not full cascaded and provided which would another potential challenge in implementing the HIS accountability framework” (Service provider from the health facility)*.

It was also added the poor leadership skill in applying different management and governance principles as another potential challenge for initiating and implementing HIS accountability framework. The respondent stated:

*“The leadership and management skill of assigned leaders is not well developed which might challenge to facilitate and cascade the implantation of HIS accountability framework” (M&E officer from zonal health department)*.

### Security treat

Political instability/insecurity is another barrier that has been explored. The respondents explained that due to security problems, they didn’t have a conducive environment to implement HIS activity monitoring, such as review meetings, supervision, and mentorships. Besides, the virtual and remote HIS monitoring and communication channels are blocked due to the interruption of the internet network. This problem affects the health professionals’ stability and psychology not to focus on the basic tasks and worry about other things. With these conditions, implementing such new initiatives will be challenging. A respondent stated:

*“In the current condition*,* the health professionals are unstable and worry about other security issues. Besides*,* due to the interruption of the internet network*,* there is no means to follow and monitor the HIS tasks face to face and virtually. Due to this*,* it might be challenging to implement the framework in this condition.” (M&E officer from zonal health department)*.

### Suggestions

Recommendations are opinions forwarded by the respondents about their views and thoughts on a specific topic. In this study, the informant’s recommendation was explored on what to do in designing an effective HIS accountability framework that will be implemented successfully.

The informants noted that the following issues must be considered during the framework design. Building skills and knowledge on HIS and leadership, making HIS the political agenda, fulfilling the basic inputs and creating a conducive work environment, engaging every concerned body, listen to the sayings, feelings, and emotions of the health professionals were among the recommended issues by the respondent.

The survey participants expressed doubts about the acceptance and effectiveness of the intervention unless it is inclusive and open to all. Therefore, involving all stakeholders in discussions about the intervention’s importance, as well as its design and execution, is essential for its success. It was stated:

*“As to me*,* most interventions have failed because they are not basing the real problem on the lower-level implementers. Therefore*,* engaging every actor and understanding their feelings and thoughts is very important for a successful intervention. If the engagement of the implementers develops the intervention*,* it will enhance the sense of ownership*,* and its adoption will be good.” (A service provider from the health center)*.

The other issue forwarded by the respondents is before coming up with a new intervention, it is good to create a conducive and ready institution to adopt and implement the intervention. They stated that before initiating the HIS accountability framework, it is good to create the skill and knowledge of implementers and leaders on HIS and leadership. This will enhance the value and attitude of the implementers toward health data. In addition, they added inputs like HIS supplies and human resource issues that must be managed. A key informant stated:

***“…****To ask about the staff’s responsibilities*,* initially*,* we need to fulfill their rights. For example*,* to command and order health professionals to correctly do the HIS activities as per the protocol and to take corrective actions*,* initially*,* we need to avail all requirements*,* including the supplies and adequate human resources.” (Head of the health center)*.

Moreover, it was mentioned that making HIS a political agenda across the health system could potentially increase awareness and focus on need for HIS accountability framework by the decision makers and politicians involved in the HIS system, thereby aiding in its successful implementation. The respondent stated:

*“In order to establish a robust and long-lasting accountability framework for HIS*,* it is essential for politicians to prioritize this issue. Attention should be directed towards advocating for the importance of health data and making it a notable political agenda.” (Head of the woreda health office)*.

## Discussion

In this study, we understood that the interviewing respondents better explained the HIS accountability framework and its advantages in improving the HIS program. In addition, we explored that even if the practice HIS accountability framework is limited, potential enablers like a conducive policy environment, HIS tools standardization, partner engagement, and human resource development were reported. Workload, leadership skill gap, lack of national HIS accountability framework guidelines, and political instability were the commonly reported barriers. It was suggested that a participatory and inclusive approach be followed in developing the HIS accountability framework.

The study showed that the interviewees explained the HIS accountability framework as a guiding and controlling system to perform the HIS tasks per the standard. The assessment showed that the participants better understood the concept of the HIS accountability framework, which is one potential opportunity to implement the intervention successfully. Familiarity and conceptual understanding of an intervention are key factors for effective implementation [24].

The study also explored the perceived advantages of the HIS accountability framework. Most respondents perceived HIS accountability as a good intervention to govern the proper implementation of HIS activities by managing behavioral problems related to HIS activities and improving health professionals’ adherence to national HIS protocols [25]. This also implies that a positive perception of the intervention’s advantage will help in its effective implementation.

However, it was also explored that the current controlling system has multiple challenges since there is no national HIS governance protocol [26, 27]. It was also stated that their practices are limited even if there is a direction to conduct HIS performance review meetings regularly, follow up, and provide supportive supervision. This makes the health system fail to manage the attitude and behavioral-related problems related to health data management. Due to this, behavioral-oriented intervention is important to manage such problems by developing HIS activity-specific terms of reference.

The study also indicated that national priority, digital expansion, standardization, partner engagement, human resource advancement in the HIS area, and hopeful progress in the HIS performance are potential opportunities to implement the HIS accountability framework effectively [28]. This implies that scaling up on these enablers would help design an effective HIS accountability framework.

The study also explored the poor skill and commitment from the leadership as potential barriers to initiating and implementing the HIS accountability framework, which aligns with different research in different parts of the country [29–31]. So, it is recommended that they work on enhancing their skills and commitment by using different modalities like capacity-building, training, and experience-sharing [32]. Frequent leadership and staff turnover also affect the sustainability of new initiatives started at the facility, which the newcomers need time to familiarize themselves with. Different investigators also explored it in different settings [33, 34]. Therefore, it is suggested that the work environment be conducive to health workers retaining them and reducing turnover.

The lack of a national HIS accountability framework is another potential barrier: facilities may not have common ground rules to design their facility-specific HIS accountability frameworks. The HIS maturity assessment done in Ethiopia also showed a gap in the practice of HIS leadership and governance, especially at sub-national and lower health system levels [28]. Besides, the acceptability and practicability of the HIS accountability framework would be challenging if the protocol didn’t have a national base. So, it is also advisable to develop a national HIS governance protocol, which would be a base and legal framework across the health system level [35].

Workload and multiple responsibilities are other important barriers: making individuals accountable for their work results might be difficult without fulfilling basic inputs like human resources [33]. Individuals becoming loaded may prioritize clinical care and health data activities like proper recording and reporting. Most of the time, staff prioritize clinical services over the data. Besides, single individuals are assigned to coordinate multiple programs at different institutions. Their focus on health data management would be limited because of their multiple responsibilities.

Lack of HIS supplies and infrastructure is also reported as another barrier, and currently, facilities are recommended to avail themselves of essential HIS supplies [33]. However, most facilities couldn’t do this due to resource constraints. With the availability of such constraints, it might also be challenging to implement the intervention effectively. So, to implement the accountability framework system effectively, these pre-intervention organizational readiness activities should be done.

Political instability or security problems would also be another barrier to effectively implementing the HIS accountability framework. It is believed that stability is a key factor for the effective implementation of interventions. However, the current political instability and conflict affect the community- and facility-level HIS activities directly and indirectly by restricting the health professionals’ movement and internet blockage.

### Strengths and limitations of the study

This study examined the practice and challenges of the HIS accountability framework from a diverse group of respondents. Understanding their perceptions is essential for designing user-focused interventions. To reduce social desirability bias, we ensured confidentiality, used neutral language, built rapport, and applied probing techniques. We also used, indirect questioning, and non-verbal observations to validate findings. However, the study did not include health extension workers, who play a key role in generating health data.

#### Recommendations

This study explored the awareness, importance, practice, enablers, and barriers. Based on the information explored, the following issues are recommended.

- Given that the Health Information System (HIS) is a key focus of the national healthcare system, it is crucial to elevate this issue to the forefront of political agendas at every level of the healthcare system.
- By improving the HIS and leadership capabilities, we can foster a more positive attitude and deeper commitment towards HIS practices.
- It is important to involve all relevant stakeholders in the development, initiation, and implementation of HIS accountability framework to promote a sense of ownership and accountability.
- Establishing a national HIS accountability framework would serve as a valuable reference for lower-level healthcare institutions.
- Availing basic HIS supplies and facilities would help to effectively implement HIS accountability interventions.

## Conclusion

This study explored the practice, enablers, and barriers of HIS accountability framework. The study revealed that most respondents better understand the concept of HIS accountability framework. However, almost all respondents stated that they didn’t have a well-organized and written HIS accountability framework.

However, it was stated that the HIS accountability framework is an essential intervention to improve the HIS program performance by managing the behavioral-related barriers of the program. Besides, the HIS accountability framework is believed to be an ideal intervention for controlling the progress of HIS activities by practicing leadership, management, and governance practices using the governing protocol. In addition, government priority, digital expansion, HIS partners’ availability, and promising progress in HIS performance were explained as potential opportunities to adopt and implement the HIS accountability framework better. The barriers explored were a poor culture of accountability practice/system, poor leadership skills, staff and leadership turnover, lack of national guidelines, workload, multiple responsibilities, poor HIS and infrastructure, and insecurity.

The respondents suggested engaging all concerned bodies while designing and implementing the HIS accountability framework. Understanding the environment’s context and health professionals’ feelings and emotions was recommended. Another suggestion was to fulfill the necessities and create a conducive work environment for the implementers before initiating the intervention.

## Electronic supplementary material

Below is the link to the electronic supplementary material.

Supplementary Material 1

Supplementary Material 2

## Data Availability

The manuscript contains a detailed summary of the identified themes, sub-themes and sample quotes presented in Table 2. Furthermore, a supplementary file, labeled as Supplementary File 01 & 02, includes the Key Informant Interview (KII) guiding tool and COREQ guidline respectively.

## Abbreviations

BSc: Bachelor of Science
DHIS2: District Health Information System
eCHIS: Electronic Community Health Information System
HCD: Human Centered Design
HIT: Health Information Technologists
HIS: Health Information System
HMIS: Health Management Information System
HSTP: Health Sector Transformation Plan
IR: Information Revolution
KII: Key Informant Interview
MPH: Masters of Public Health
M&E: Monitoring and Evaluation
PMT: Performance Monitoring Team
RHB: Regional Health Bureau
ZHD: Zonal Health Department
