# Supplementary material for: Practice, enablers and barriers of health information system accountability framework in Northwest Ethiopia 2023
Source: BMC Med Inform Decis Mak. 2025 Mar 3;25:107. doi: 10.1186/s12911-025-02942-8 (PMC11877924; doi:10.1186/s12911-025-02942-8)
Supplement: Supplementary file 1 — Supplementary Material 1 [file 12911_2025_2942_MOESM1_ESM.docx]

# **Title: - The Practice, Enablers and Barriers of Health Information System Accountability Framework in Northwest Ethiopia, 2023**

1. **General information**

Name of Interviewer: _________________ Date: _____________________________

Time discussion started: _______________ Time Interview ended: ________________

Name of district: _______________________ Name of Institution: __________________

1. **Demographic characteristics of participants in the discussion**

| No | Code of Participant | Sex | Age | Years on the current position | Years of experience | Educational Level | Position |
| --- | --- | --- | --- | --- | --- | --- | --- |
| 1 |  |  |  |  |  |  |  |

1. **KII guiding questions**

| **No** | **Questions** | **Probing** |
| --- | --- | --- |
|  | How do you explain the healthcare accountability framework? | Probing:   - Understanding of health accountability framework/Prior information on the concept - Source of information (written documents/job description/directives/guidelines/peers/supervisors) - How are those frameworks presented? (written/unwritten) - Its importance for the individual, program, and institutional performance |
|  | How do you explain the HIS accountability framework? | Probing:   - Understanding of health accountability framework/Prior information on the concept - Source of information (written documents/job description/directives/guidelines/peers/supervisors) - How are those frameworks presented? (written/unwritten) - Its importance for the individual, program, and institutional performance |
|  | How do you feel about the importance of HIS accountability framework for health data quality and use? | Probing:   - Perceived advantage/disadvantage of practicing HIS accountability framework (performance, motivation, accountability, satisfaction, data quality, data use, service delivery) |
|  | Does your institution implement any system/framework to govern HIS-specific activities (HIS accountability system) using tools, Terms of Reference, Directives, or procedures? | Probing:   - How the document was prepared and framed, who were the participants, when it was started, and what did the contents and procedural activities seem like? - Did the system apply practically? (Feedback, warning, rewarding/recognition? - What is the reflection you got from the action? |
|  | How do you explain the opportunities/enablers for implementing the HIS accountability framework? | Probing:   - Leadership engagement and commitment - Staff acceptance and capacity - System support/legal framework - National priority |
|  | What do you think about the barriers to initiating and implementing HIS accountability framework? | Probing:   - Leadership engagement - System/legal procedural document - Technical skill - Staff engagement - Multiple responsibility/assignment/position - Workload……. |
|  | What is your opinion or suggestion on designing and implementing the HIS accountability framework? | Probing   - Participatory and engagement - Inclusiveness - Preparation - Fulfillment |
